# 

**Published:** 1892-11

**Authors:** 


### SOCIETY MEETINGS.

### IOWA STATE VETERINARY MEDICAL ASSOCIATION.

The Iowa State Veterinary Medical Association will hold its fifth annual session at Marshalltown, Iowa, November 10th and 11th. An attractive programme is offered, which, in addition to the routine business, includes a paper on "Fistula," by M. H. Reynolds; "Sclerostoma Tertracanthum," by G. L. Buffington, Mt. Pleasant, Iowa; "Intestical Derangement," by A. R. Williams, Glenwood, Iowa; "Spaying," by J. G. Kennedy, West Union, Iowa; "Disinfectants in Veterinary Practice," by W. B. Niles, Ames, Iowa; paper by W. L. Williams, Purdue University, Indiana; notes of cases peculiar to Arizona, by J. C. Norton, Phoenix, Arizona.

### ILLINOIS STATE VETERINARY MEDICAL ASSOCIATION.

The annual meeting of the Illinois State Veterinary Medical Association will be held in the Sherman House, Chicago, Ill., November 16th and 17th. The Secretary informs us that an interesting programme has been arranged.

